# An Improved Colonoscopy Preparation Method and its Acceptability by Patients

**DOI:** 10.1155/DTE.1.141

**Published:** 1995

**Authors:** Masanori Tsuruoka

**Affiliations:** Tsuruoka Clinic 54-3 Ryoujihouji Higashinakatomi Aizumi-cho Itano-gun Tokushima 771-12 Japan

## Abstract

The author presents an improved method of preparation for colonoscopy that involved no dietary limitation on the patient until the day of the examination and that was shown by a randomized questionnaire evaluation to earn good patient tolerance and acceptance. Patients were given 10 mg of cisapride and 75 mg of sodium picosulfate before sleep on the day preceding the examination, and 50 g of magnesium citrate powder (MP) in 1,200 mL lukewarm water before the examination. It was divided into 600-mL portions and ingested slowly during two 30-minute periods. Ninety-five percent of patients classified the taste of a magnesium citrate powder laxative as palatable in the questionnaire given immediately after the procedure. Concerning the quantity, 79.4% replied that it was tolerable, 17.3% considered it somewhat excessive, and 3.3% replied that it was barely tolerable. No patient classified it as intolerable. Symptoms after taking laxatives and lukewarm water such as abdominal pain, nausea and abdominal fullness were observed in 3.8%, 4.4% and 5.6%, respectively, whereas there were no symptoms in 79% of patients. Body weight and serum K level showed a tendency to decrease, whereas the serum Mg level showed an increase before and after colonoscopy. The quality of colonic cleansing evaluated by colonoscopy was excellent, good, or fair in a total of 93.3%. No adverse effects were observed. It was concluded that this method is a clinically beneficial and well-tolerated preparation for colonic examinations.


*Diagnostic and Therapeutic Endoscopy*, 1995, Vol. 1, pp. 141–145
Reprints available directly from the publisher
Photocopying permitted by license only

© 1995 Harwood Academic Publishers GmbH
Printed in Malaysia

### An Improved Colonoscopy Preparation Method and its Acceptability by Patients

MASANORI TSURUOKA

*Tsuruoka Clinic, Tokushima 771-12, Japan*

*(Received January 27, 1994; in final form April 20, 1994)*

The author presents an improved method of preparation for colonoscopy that involved no dietary limitation on the patient until the day of the examination and that was shown by a randomized questionnaire evaluation to earn good patient tolerance and acceptance. Patients were given 10 mg of cisapride and 75 mg of sodium picosulfate before sleep on the day preceding the examination, and 50 g of magnesium citrate powder (MP) in 1,200 mL lukewarm water before the examination. It was divided into 600-mL portions and ingested slowly during two 30-minute periods. Ninety-five percent of patients classified the taste of a magnesium citrate powder laxative as palatable in the questionnaire given immediately after the procedure. Concerning the quantity, 79.4% replied that it was tolerable, 17.3% considered it somewhat excessive, and 3.3% replied that it was barely tolerable. No patient classified it as intolerable. Symptoms after taking laxatives and lukewarm water such as abdominal pain, nausea and abdominal fullness were observed in 3.8%, 4.4% and 5.6%, respectively, whereas there were no symptoms in 79% of patients. Body weight and serum K level showed a tendency to decrease, whereas the serum Mg level showed an increase before and after colonoscopy. The quality of colonic cleansing evaluated by colonoscopy was excellent, good, or fair in a total of 93.3%. No adverse effects were observed. It was concluded that this method is a clinically beneficial and well-tolerated preparation for colonic examinations.

**KEY WORDS:** colonoscopy, colon preparation, no dietary restriction

### INTRODUCTION

Considerable improvements have been made in recent years in colonoscopy. Currently, there are three methods of colon cleansing that are commonly used and that have serious effects on the outcome. They are the Brown method (1), which requires prescribed diets on the previous day, the Davis method (2), which is a peroral bowel cleansing technique requiring an ingestion of a large quantity of polyethylene glycol electrolyte lavage solution (PEG); and the Vanner method (3), in which a small volume of sodium phosphate solution (NaP) is given. In addition, there are modifications of these methods (4,5).

The Brown method, however, has some problems including hunger owing to dietary restrictions, sleeping disturbance owing to diarrhea on the preceding day, and procedural complexities. In addition, this method often fails to produce satisfactory results in cleansing the distal colon, and it is difficult to insert the fiberscope smoothly at colonoscopy owing to dryness of the colon wall. The Davis method, by contrast, is effective in cleansing the total colon, and the fiberscope is readily inserted. However, the large quantity of the ingested water requires a longer time for absorption and a large quantity of residual colon fluid and foaming in the colon occasionally makes colonoscopic observation difficult. Furthermore, patients are frequently distressed by this method because of abdominal fullness and chills after ingestion of 2 to 3 L of water and the unpleasant taste of the solution. The cathartic action of the NaP results largely from its osmotic properties, and given its small volume yet large resulting effluent there are concerns about potential intravascular volume depletion. The taste of NaP solution is saltier than PEG. To overcome these problems, the author developed a new method.

Address for correspondence: Masanori Tsuruoka, M.D., Tsuruoka Clinic 54-3 Ryoujihouji, Higashinakatomi Aizumi-cho, Itano-gun, Tokushima 771-12, Japan.

### METHODS

The subjects were 180 patients (aged 16 to 83 years) who were scheduled for colonoscopy at our hospital. (Those with serious complications, such as heart disease, renal dysfunction, and liver cirrhosis were excluded.) Because no restrictions were placed on the diet the previous day, the patients could consume ordinary foods.

Before ingesting magnesium citrate powder (MP) (Horii Pharmaceutical Industries, Ltd., Osaka, Japan), the patient was instructed to take 75 mg of sodium picosulfate and 10 mg of cisapride before retiring the night before to soften the fecal texture and ease the transit of the intestinal contents to the lower intestinal tract.

On the day of examination, the patient was allowed no food, and 4 hours and 3.5 hours before examination, was instructed to slowly drink, in two 600-mL portions, 1,200 mL of lukewarm water in which 50 g of MP had been mixed thoroughly and which had a flavor suggestive of fruit juice.

A survey (via questionnaire) was administered at random to 100 of the test subjects (aged 18–78 years) to determine the degree of discomfort associated with the taste and quantity of liquid they were required to imbibe. Because the taste of this fluid plays a significant role in its acceptance, the survey was designed to compare and rate the flavors and acceptability of the liquid prepared by us, PEG, and the NaP solution developed by Vanner *et al.* by choosing one of three categories.

To evaluate the safety of the method presented here, the following clinical parameters were recorded in all cases on the day before and immediately before the examination: body weight, blood pressure, hematocrit, erythrocyte count, leukocyte count, serum electrolyte contents (Na, K Cl, Mg and Ca), creatinine level, blood urea nitrogen, and blood urea acid.

The residual fluids in the colon shift with postural changes. Therefore the overall state of colonic evacuation at endoscopy was rated at one of the following five levels instead of by examining various specific anatomic sites within the colon:

Level 1—Excellent; no fecal residues are present, and the fluid remaining in the colon is almost clear.

Level 2—Good; no fecal residues are present, but the remaining fluid is turbid.

Level 3—Fair; small amounts of fecal matter remain in some sections, but the mucosal surface becomes clear by repeated irrigation and suction by fiberscope.

Level 4—Poor; a large amount of fecal matter remains, and irrigation and suction are not sufficient to break it up.

Level 5—Inadequate; hard or semisolid fecal matter remains, preventing the passage of the colonofiberscope.

### RESULTS

### Evaluation of the Pretreatment by the Test Subject

*Quality, flavor, and ease of swallowing the cleansing fluid*
In response to the questions on the flavor and volume of the liquid to be ingested, all patients stated that MP was easy to swallow. As for the bulk of the fluid, 79.4% stated that they experienced no discomfort, 17.3% considered the volume to be slightly excessive, 3.3% reported that they suffered a degree of discomfort, and none found it impossible to consume the required quantity. The preparation was described as "easy to ingest" by 95%, whereas only 5% reported difficulty in swallowing it (Table 1).

According to the evaluation by the 100 test subjects of the fluid used in the present procedure, PEG, and the NaP liquid, MP solution was the easiest to drink, followed by PEG, and the NaP liquid (in descending order of acceptance).

*Symptoms following ingestion of cathartics*
The incidence of symptoms developing after ingestion of the cathartics included abdominal pain (3.8%), nausea (4.4%), and abdominal fullness (5.6%). None reported vomiting or chills, and 79% did not experience any symptoms (Table 2).

### Changes in Body Weight and Blood Pressure and Results of Hematologic Tests and Blood Chemical Analyses Before and After Colonoscopy

Table 3 shows body weights and the results of hematologic tests and blood chemical analyses before and after the colonoscopic procedure. The body weight decreased

**Table 1** Impression of patients on the amount of fluid intake and the taste of the laxative (magnesium citrate powder)

| *Item* | *Impression* | *Results (%)* |
|---|---|---|
| Quantity of drinking water | Tolerable | 79.4 |
| | Somewhat excessive | 17.3 |
| | Barely tolerable | 3.3 |
| | Intolerable | 0.0 |
| Taste of laxative | Palatable | 95.0 |
| | Nonpalatable | 5.0 |

**Table 2** Symptoms after taking laxatives

| *Symptom* | *Results (%)* |
|---|---|
| Abdominal pain | 3.8 |
| Nausea | 4.4 |
| Abdominal fullness | 5.6 |
| Vomiting | 0.0 |
| Anal pain | 7.2 |
| Chill | 0.0 |
| No symptom | 79.0 |

**Table 3** Comparison of body weight and hematologic, and biochemical changes before and after colonic examination

| | *Before preparation* | | | *After preparation* | | |
|---|---|---|---|---|---|---|
| | *Mean* | *Standard deviation* | | *Mean* | *Standard deviation* | |
| Weight (kg) | 59.5 | ± | 11.1 | 58.8 | ± | 11.1 |
| Blood pressure (Hg) | 124.2 | ± | 16.2 | 123.1 | ± | 15.5 |
| Erythrocyte ( × $10^4$) | 434.2 | ± | 45.5 | 431.4 | ± | 47.8 |
| Leukocyte | 5,741.7 | ± | 1,198.1 | 5,985.0 | ± | 1,245.3 |
| Hematocrit (%) | 39.2 | ± | 3.3 | 39.3 | ± | 3.1 |
| BUN (mg/*dl*) | 14.3 | ± | 3.6 | 12.9 | ± | 3.4 |
| Creatinine (mg/*dl*) | 0.9 | ± | 0.2 | 0.9 | ± | 0.2 |
| UA (mg/*dl*) | 5.0 | ± | 1.5 | 5.1 | ± | 1.4 |
| Na (mEq/L) | 140.0 | ± | 3.7 | 141.3 | ± | 2.3 |
| Cl (mEq/L) | 103.5 | ± | 2.5 | 103.3 | ± | 2.5 |
| Ca (mEq/L) | 4.5 | ± | 0.4 | 4.5 | ± | 0.2 |
| K (mEq/L) | 4.1 | ± | 0.4 | 3.9 | ± | 0.6 |
| Mg (mEq/L) | 2.0 | ± | 0.2 | 2.2 | ± | 0.2 |

**Table 4** Cleansing effectiveness assessed with colonoscopy

| *Effectiveness* | % |
|---|---|
| Excellent | 16.7 |
| Good | 44.4 |
| Fair | 32.8 |
| Poor | 6.1 |
| Total | 100.0 |

slightly in most (from 59.5 ± 11.1 kg to 58.8 ± 11.1 kg). Blood pressure showed no significant change. There were no changes between "before" and "after" colonoscopy in erythrocyte and leukocyte counts, hematocrit and serum calcium, and Na and Cl levels.

The serum K level showed a tendency to decrease (from 4.1 ± 0.4 mEq/L to 3.9 ± 0.6 mEq/L), whereas the serum Mg level showed a significant increase (from 2.0 ± 0.2 mEq/L to 2.2 ± 0.2 mEq/L, $p<0.01$), but both were still within the normal ranges.

### The Extent of Cleansing Determined by Colonoscopy

Table 4 shows the results of colonoscopic evaluation of colonic cleansing following the application of the present procedure. The sum of the excellent, good, and fair ratings was 93.9%. The mean amount of retained fluid solution during colonoscopy was from 30 to 100 mL.

### DISCUSSION

Many attempts have been made to improve colonic preparations and thus enhance the diagnostic accuracy of colonoscopy. However, there are still many problems to be solved regarding acceptability by the patient. The optimum preparation for most patients before a colonoscopic procedure will be one that can be performed at home and meets the following conditions: pleasant flavor of the fluid to be ingested; a fluid volume that is not excessive; cleansing produced simply, safely, and rapidly; and a normal diet on the day before the examination.

In 1961, Brown (1) introduced a total intestinal irrigation procedure in which salt and contact cathartics were administered while the patient was on a low-residue, low-fat diet. Obviating the use of an enema, it was considered to be a revolutionary method of preparation. However, the dietary restrictions imposed by this procedure caused calorie insufficiency, and the patient often suffered from hunger. The procedure was complex, often interfering with the daily activities on the previous day. Furthermore, diarrhea on the night before the scheduled test often deprived patients of sleep. In some cases, the cleansing effect was insufficient in deep crypts of the colon and it was difficult to insert the fiberscope smoothly owing to the dryness of the colon wall.

In 1980, Davis *et al.* (2) developed a pretreatment procedure using polyethylene glycol. Its intestinal cleansing effect was excellent. The procedure allows consumption of normal meals until the day before the examination, a marked improvement over Brown's procedure. However, the original method by Davis required 3 to 4 L of PEG, which is salty and difficult to ingest in a relatively short time until the patient passes clear fluid. This was poorly tolerated and not readily accepted by patients. Although the procedure produced an excellent colonic cleansing effect, a large volume of fluid remained (6,7).

Villen and Rytkonen (1990) reported a study using only 1.5 L of PEG, but their method required some dietary restrictions on the day before the colonoscopy, and the inclusion of PEG meant a salty taste (8). In addition, there is a modification of this method with improved taste and less net water (4,5). Furthermore, occasionally a foaming phenomenon provoked in the intestines resulted in extra time being required for suction during colonoscopy and frequent interference with the visual field (9–11).

Vanner *et al.* (1990) proposed a procedure in which 45 g of NaP fluid was given twice. This method was more acceptable for patients because it only required ingestion of a small amount of liquid; however, it was associated with shortcomings such as hospitalization, restrictions on the evening meal the night before, the taste of the fluid, which was even saltier than PEG, and a large volume of remaining fluid. Furthermore, it was up to the individual patient to replenish fluid despite exacerbated diarrhea, thus exposing the patient to the hazard of dehydration.

The author set out to develop a preparatory procedure for colonoscopy that would eliminate the above-described problems and ease discomfort to the patient and developed a method that permits consumption of a normal meal on the day before the examination (12).

The present study introduces a simplified procedure in which the author's previous procedure was modified by adding cisapride, an agent to enhance gastrointestinal motility, reduce the volume of the fluid to be consumed and which is given in two divided dosages. It has been reported that with the procedure of Davis *et al.*, and Vanner *et al.*, a large volume of fluid remained in the colon (3,7). In the present study the amount of remaining fluid in the colon was small.

The quantity of absorption of the retained fluid during colonoscopy was little by the present method. The patient's acceptability of the procedure improved markedly. Many experienced none of the symptoms that had been associated with earlier procedures. None reported chills and vomiting, which sometimes occurred when the PEG procedure (and its modification) was employed, in which a patient is required to consume a large quantity of cold liquid, chilled to make it more palatable. The fluid used in the present procedure is free of any unpleasant flavor and the patient can consume it at a lukewarm temperature. These differences appear to explain the absence of the development of chills and vomiting in those undergoing this new regimen.

Flavor has a significant effect on the patient's acceptance. Both PEG and NaP fluid are very salty and unpalatable. The MP solution, when dissolved in lukewarm water, produces a flavor similar to that of fruit juice. It was ingested with relative ease thus improving the acceptability of the present procedure. According to the results from our questionnaire, 95% stated that they ingested the specified quantity of the fluid without experiencing any distaste or discomfort. Thus the taste was well accepted by the test subjects. Only 5.0% reported that they ingested it with effort. All were able to consume the specified amount, leading to a successful test.

Although some innovations have been added to the PEG procedure to improve the taste of the fluid, including administration of orange juice (13), addition of saccharine and lemon essence (11), removal of sulfates from PEG (4), and reductions in the Na and sulfate contents (5), the PEG procedure does require the intake of a large quantity of fluid, and many patients complain about abdominal fullness during the test.

Few complained of fullness during the present improved procedure. It is believed that the difference was probably a result of the reduction in volume of the fluid to 1,200 mL, which was divided into 2 600-ml portions and ingested slowly on two separate occasions.

Loss in body weight, electrolyte imbalance, and dehydration may develop owing to exacerbated diarrhea associated with the preparation for colonoscopy. Therefore body weight, blood pressure, and the results of hematologic tests and blood chemical analyses recorded before and after the pretreatment were compared. Body weight and the serum K level showed a tendency toward reduction, whereas the serum Mg level rose significantly. However, none of these data exceeded the normal ranges. No patient exhibited a rise in hematocrit levels. The reduction in body weight was believed to be caused by evacuation of the intestinal content and fasting on the day of the examination. In those cases in which the colonoscopic evaluation revealed excellent to fair colon cleansing, the vascular and mucosal surfaces could be closely observed through repeated suction and irrigation. In the present improved procedure, 93.9% of the patient population showed excellent to fair cleansing results, permitting a complete endoscopic examination.

The improved preparation procedure for colonoscopy described in this report was found to be safe and to cause little discomfort and could be accomplished at home. Self-administration has an added advantage: it saves health services manpower.

As described above, the procedure for colonoscopic preparation designed by the author requires fasting only on the day of examination. The flavor of the fluid is satisfactory, and its volume is relatively small. The patients do not suffer from dehydration. Thus the clinical efficacy of this procedure is believed to be significant.

### ACKNOWLEDGMENTS

This paper was presented in part at the 11th Colorectal Mass Survey Research Meeting on July 27, 1991, at

Tokyo, Japan. The author gratefully acknowledges Prof. J. Patrick Barron and Ms. Chiharu Sakata of the International Medical Communications Center, Tokyo Medical College, for their translation and repeated revision of the manuscript.

### REFERENCES

1. Brown GR. A new approach to colon preparation for barium enema. Univ Michigan M Bull 1961; 27:225–238
2. Davis GT, Santa Ana CS, Morawski SG, et al. Development of a lavage solution associated with minimal water and electrolyte absorption or secretion. *Gastroenterol*, 1980; 78:991–995
3. Vanner SI, MacDonald PH, Paterson WG, et al. A randomized prospective trial comparing oral sodium phosphate with standard polyethylene glycol-based lavage solution (PEG) in the preparation of patients for colonoscopy. *Am J Gastroenterol*, 1990; 85:422–427
4. DiPalama JA, Marshall JB. Comparison of a new sulfate-free polyethylene glycol electrolyte lavage solution versus a standard solution for colonoscopy cleansing. *Gastrointest Endoscop*, 1990; 36:285–289
5. Tomlinson TL, DiPalma JA, Mangano FA. Comparison of a new colon lavage solution (Golytely-RSS) with a standard preparation for air-contrast barium enema. *Am J Roentgenol*, 1988; 151:947–950
6. Shimizu S, Mizuma Y, Ogawa M et al. Evaluation of polyethylene glycol electrolyte lavage solution in preparation for colonoscopy. *Gastroenterol Endoscop*, 1987; 29:3080–3086.
7. Goldman I, Reichelderfer M. Evaluation of rapid colonoscopy preparation using a new gut lavage solution. *Gastrointest Endoscop*, 1982; 28:9–11
8. Villen M, Rytkonen M. PEG preparation for colonoscopy: 1.5 liters is enough for outpatients. *Endoscopy*, 1990; 22:157–202.13.
9. Hangartner PJ, Münch R, Meier J, et al. Comparison of three colon cleansing methods: evaluation of a randomized clinical trial with 300 ambulatory patients. *Endoscopy*, 1989; 21:272–275.
10. Matsumoto T, Murase T, Ka K et al. Evaluation of pretreatment for colonoscopy in aged examinees. *Gastroenterol Endoscop*, 1989; 31:490–495
11. Seki T, Tani K, Okuhira M et al. Dimethylpolysiloxane as a deforming agent in gut lavage solution for colonoscopy. *Gastroenterol Endoscop*, 1990; 32:2435–2438
12. Tsuruoka M. Preparation for colonoscopic examination without dietary restriction on the previous day. *Gastroenterol Endoscop*, 1990; 32:941–948
13. Hangartner P. J., Münch R., Meier J., et al. Comparison of three colon cleansing methods: evaluation of a randomized clinical trial with 300 ambulatory patients. *Endoscopy*, 1989; 21:272–275

